# A global dataset of carbon pumping by the world’s largest tropical rivers

**DOI:** 10.1038/s41597-024-03201-7

**Published:** 2024-04-13

**Authors:** Luca Salerno, Fabio Giulio Tonolo, Carlo Camporeale

**Affiliations:** 1 DIATI Department of Environment, Land and Infrastructure Engineering, Politecnico di Torino, Turin, 10129 Italy; 2 DAD Department of Architecture and Design, Politecnico di Torino, Turin, 10129 Italy

**Keywords:** Environmental impact, Hydrology

## Abstract

The eco-morphodynamic activity of large tropical rivers interacts with riparian vegetation causing implications for the carbon cycle within inland waters. Through a multi-temporal analysis of satellite data spanning the years 2000–2019, we analyzed rivers exceeding 200 m in width across the tropical regions, revealing a Carbon Pump mechanism driving an annual mobilization of 12.45 million tons of organic carbon. The study identifies fluvial eco-morphological signatures as proxies for carbon mobilization, emphasizing the link between river migration and carbon dynamics. To enhance accessibility, our results are encapsulated in a visually compelling WebGIS application, offering a comprehensive understanding of the eco-geomorphological influences on the global carbon cycle within large tropical rivers. Our findings are instrumental in determining the carbon intensity of future hydropower dams, thereby contributing to informed decision-making in the realm of sustainable energy infrastructure. This study elucidates the intricate relationships that govern the nexus of tropical river dynamics, riparian ecosystems, and the global carbon cycle.

## Background & Summary

The traditional perception of river networks as passive and unchanging conduits for water and sediments, solely transporting them from their source to the oceans, has undergone substantial reevaluation in recent decades^1,2^. Rivers represent intricate and dynamic systems in which carbon is actively generated, transported, transformed, and stored in diverse forms, profoundly impacting the global carbon cycle across varying spatial and temporal scales^3–5^.

The assessment of carbon fluxes within the inland water carbon cycle has seen progressive refinement, elucidating our current comprehension of carbon exchange with the soil, atmosphere, and oceans. Specifically, estimates of terrestrial carbon input from wetland and riparian ecosystems remain subject to uncertainty and likely underestimation due to recognized and unidentified gaps^6^. Although the carbon flux from terrestrial ecosystems to the oceans is acknowledged as a pivotal pathway of the carbon cycle, the role of river dynamics in mobilizing carbon stored in extensive woodlands has largely been disregarded^7^.

During extreme events or river migration, water streams recruit a substantial amount of wood from the riparian zone. However, the destiny of this material remains inadequately understood. The classical perspective of the River Continuum Concept^8^ posits that Large Woods (LW) originating from floodplains undergo fragmentation, decomposition, and re-emission through outgassing. Nevertheless, multiple studies have provided evidence suggesting that once the river channel recruits LW, they can persist and remain buried within the alluvium for exceptionally long periods^9,10^. The storage of riverine sediment plays a crucial role in biogeochemical cycling^5^, as a significant portion of organic carbon from the biosphere is retained in terrestrial reservoirs for thousands of years prior to its eventual ultimate deposition in marine basins^11^.

The conventional approach used to determine global carbon export estimates as particulate organic carbon (POC) overlooked the inclusion of this coarse material^5^. While various techniques are currently under development to track and quantify the transportation of woody material by rivers^12–14^, these methods are predominantly constrained to local scales, and a comprehensive evaluation of global wood export remains absent.

**Fig. 1:**
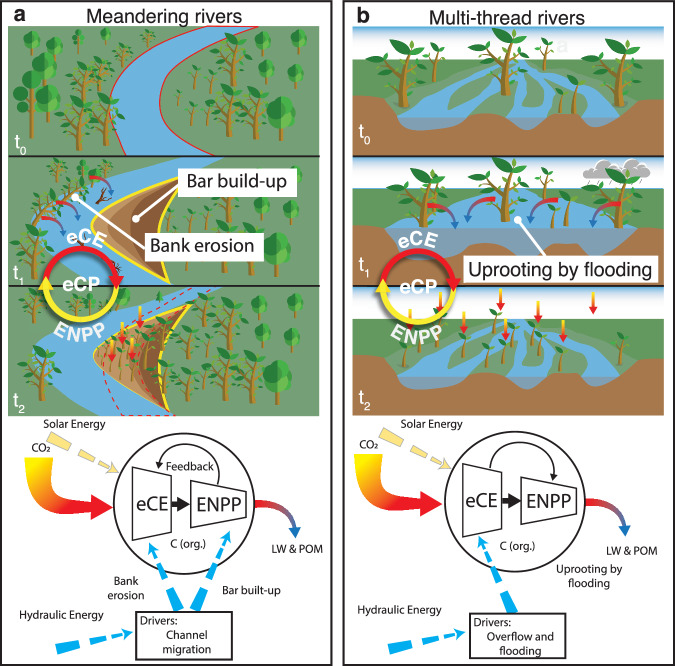
The functioning of the ecomorphodynamic Carbon Pumping (eCP) mechanism. (**a**) In meandering rivers, channel-migration-driven capture of woody biomass is exported from the outer bank into the stream (eCE). Young biomass then colonizes the inner newly deposited point bar, so driving further CO_2_-fixation from the atmosphere (ENPP) and promoting further river migration (feedback effect). Hydraulic energy (dashed blue arrows) drives morphodynamics and channel migration, while solar energy (dashed yellow arrows) drives the consequent CO_2_ fixation from the atmosphere. (**b**) In multi-thread rivers, extreme hydrologic events cause a reorganization of the floodplain, exporting biomass from bars, banks, and islands into the stream (eCE). Young vegetation colonizes the newly available spots driving further CO2 fixation from the atmosphere (ENPP). Hydraulic energy (dashed blue arrows) drives the overflow events, while solar energy (dashed yellow arrows) drives the consequent CO_2_ fixation from the atmosphere. In both cases, the output of the pump is the mobilization of LW and POM, which is eventually stored in river channel sediments or in oceans.

As emphasized by ref. ^7^, the mobilization of carbon is initiated through a two-step carbon pumping mechanism (Fig. 1) that comprises Eco-Morphodynamic Carbon Export (eCE) and Enhanced Net Primary Production (ENPP). eCE refers to the carbon export from floodplains, whereas ENPP consists of enhanced C-fixation promoted by vegetation encroachment on bare riparian areas generated by the morphodynamic activity. We, therefore, define the eCP as the combination of these two processes, that work in cascade, and that are mainly energized by channel migration in meandering rivers and by overflow and flooding in multi-thread rivers (Fig. 1).

Rivers periodically rework the floodplains, removing vegetation through uprooting or bank erosion. This vegetation recruitment by fluvial dynamics represents a pathway through which organic carbon stored in the terrestrial ecosystem enters the inland waters. The flow of organic carbon that reaches rivers and travels through the fluvial network, can result from soil leaching (DOC or POC), or from this morphodynamic-driver recruitment. While the role in the carbon cycle of the first component is well recognized (although not constrainedly assessed), the second component is often implicitly assumed to be rapidly degraded and mineralized. We argue that it is a misconception to assume that all woody input is decomposed and reduced to micrometric size during the transit time in the fluvial system, and hence solely contributes to the fine component. The degradation time of the wood material^15–18^ is very often greater than the time it takes to reach the oceans. This is true both if the wood material leaves rivers and floodplains in a few months and when it is buried (and kept in anoxic conditions that protect it from degradation) and re-mobilized many times before reaching the ocean. Unlike the fine or dissolved fraction, the carbon contained in this coarse material can reside for a long time in floodplains or oceanic sediments before being mineralized and returning to the atmosphere.

Moreover, river dynamics are fundamental in the growth and development of floodplain forests. The morphodynamic-driven vegetation recruiting generates bare fertile riparian areas wherein new vegetation can rapidly grow. This continuous vegetation rejuvenation process influences the net primary production of the floodplain forests maintaining the system at an intermediate highly productive stage. Like the biological carbon pump^19^, whereby phytoplankton net production and its ultimate marine fall drive carbon from the atmosphere to ocean interior and seafloor sediments, we conjecture that photosynthetic fixation by riparian vegetation, the recruitment of LW, its transport, and burial, fit together in an integrated nexus in which rivers drive a carbon pump^7^ from the atmosphere to long-term stocks (i.e. floodplains and ocean).

Our study underscores the significant influence of river morphology on carbon fluxes within terrestrial systems, river corridors, and the atmosphere. Our geodatabase^20^ focused on tropical regions for two primary reasons. First, the processes under investigation are driven by the interactions between river morphological activities and riparian vegetation. Such interactions manifest in rivers where fluvial connectivity facilitates the exchange of water, sediment, and nutrients between floodplains and the river itself. Conversely, regulation and fragmentation constrain rivers’ capacity to flow freely, impacting interactions between rivers and terrestrial ecosystems and altering or inhibiting eco-morphodynamic Carbon Export (eCE). Moreover, due to the limited temporal availability of multispectral satellite data (spanning less than half a century), it was imperative to examine areas where processes evolve rapidly–specifically, where vegetation response to river disturbance is exceptionally swift and detectable within the available data timeframe. The tropics emerged as an ideal study region for these considerations. In tropical regions, large rivers persist in their pristine state, unaltered by human activity, and feature dense, highly productive vegetation covering the floodplains. Secondly, the tropics warrant special attention due to the threat posed to river connectivity by the construction of a substantial number of new dams (351 large new dams are planned in the Amazon, La Plata, and Andean foreland basins alone). As elucidated in the study by ref. ^7^, tropical countries urgently require comprehensive watershed management interventions to mitigate the impact of freshwater exploitation on the global carbon cycle.

In the present geodatabase^20^, we provide a comprehensive global-scale assessment, expanding upon the findings of a recent Neotropics-focused investigation^7^, to include regions in Africa and Asia/Oceania. The objective was to evaluate the annual recruitment of carbon in the form of Large Woods (LW) during the period 2000–2019 across 162 major tropical rivers within 402 specified regions of interest (ROIs). Collectively, these rivers annually exported an estimated amount of 12.38 ± 0.96 million tons of carbon in the form of recruited riparian biomass. This recruitment contributes to the formation of a carbon sink, primarily through the deposition and burial of LW in the floodplain sedimentary compartment but with potential extension to the ocean.

Through the analysis of the present geodatabase^20^, we have identified four discernible signatures of fluvial eco-morphological activity that serve as proxies for assessing the carbon mobilization potential associated with river dynamics. These signatures provide valuable insights into the relationship between river processes and the efficient mobilization of carbon within fluvial systems. A visualization of the main results of this study is encapsulated in a WebGIS application.

In conclusion, this work analyses the effect that a set of specific geomorphic disturbances (the ones induced by river dynamics) has on the carbon cycle. This opens the way to extend this concept to other natural processes that cause the removal of mature vegetation, preserve the carbon contained in that vegetation from rapid re-emission in the atmosphere and force the ecosystem to a juvenile high productive stage. As argued by ref. ^21^, areas where vegetation communities are disturbed frequently by high geomorphic activities, can experience an enhancement of NPP. That is due to the development of early-successional forest ecosystems that populate the area after disturbance and can sequestrate carbon rapidly. In part, this enhancement on NPP can be also due to the time gap between vegetation succession and full recovery of soil microbiological communities, which limits the decomposition of organic matter in the early phase of recovery^5^. Those phenomena are still poorly understood and further investigations need. We further remark that a comprehensive assessment of the role of river dynamics on the carbon cycle should be integrated with the analysis of the autotrophic biomass, and thus ecosystem metabolism, and metabolic regimes as suggested by ref. ^22^.

Since eCP (eCE and ENPP) is strictly correlated with sediment supply and river migration, alterations in lateral erosion, uprooting, and overflow in natural rivers negatively influence this process. Furthermore, increasing development and changes in climate and land use are bringing severe impacts on streams, which are increasingly being regulated thereby altering the natural migration of the river, the flow pattern, and the intermittency of dry and wet periods in floodplains.

These data present a valuable resource for delving deeper into the influence of eco-geomorphology in large tropical rivers on the global carbon cycle. Moreover, the insights derived from this analysis hold significance for shaping future water management policies, specifically concerning carbon, in these river systems. As highlighted by ref. ^7^, accurately quantifying carbon mobilization driven by river migration is a critical determinant in establishing the carbon intensity of forthcoming hydropower dams.

## Methods

We conducted the analysis on tropical rivers wider than 200 m, covering the period from 2000 to 2019, previously classified as “free-flowing” with minimal human disturbance according to ref. ^23^. This investigation yielded a geodatabase consisting of 162 large rivers encompassing 402 regions of interest (ROI). The total length of these rivers reached 108,000 km, covering an overall analyzed area of 403,000 km^2^, of which approximately 62,000 km^2^ represents active wet surfaces (see *River filtering* and *ROI definition*).

To examine the impact of river dynamics on vegetation, we employed satellite remote sensing analysis using the freely accessible Google Earth Engine platform^24^. Specifically, our focus was on the vegetation loss within the river corridors. By employing a probabilistic classification mapping technique (described in *Probabilistic classification model*), we estimated that the affected area during the period from 2000 to 2019 was approximately 17,693 km^2^, equivalent to a mean annual forest loss of 931 km^2^/year.

### Definition of procedure for eCE assessment

In this section, we present a schematic representation of the framework used in this study to quantify the recruitment of carbon stored in standing riparian vegetation induced by large tropical rivers. Further details of the procedure are described in the next sections. The eCE assessment procedure is based on the analysis of satellite products in order to identify the portions of floodplain forest recruited by river morphodynamic activities, and thus estimate the organic carbon contained in the biomass exported from terrestrial ecosystems to inland waters.

The identification of suitable rivers for analysis entails a rigorous selection process, guided by specific criteria:

- *Visual Inspection of Landsat Imagery and CSI Index Analysis*: Landsat imagery is subjected to meticulous visual examination, coupled with an in-depth analysis utilizing the Channel Stability Index (CSI) developed by ref. ^23^. This process aids in the identification and exclusion of rivers impacted by anthropogenic influences.
- *Assessment of Main Channel Width Relative to Mean Annual Discharge*: To ensure compatibility with the spatial resolution of the datasets utilized, an evaluation of the main channel width in relation to mean annual discharge is carried out, as outlined by ref. ^25^.

Through this rigorous selection process, we successfully identified a substantial network of rivers spanning approximately 108,000 kilometers across tropical regions, with a minimum width threshold of 200 meters, characterized by preserved connectivity and consequential hydrodynamic behavior.

Each selected river was divided into regions of interest (ROIs). The ROIs, which represent the elementary units for the analysis, were defined through longitudinal and lateral boundaries in order to identify floodplain areas characterized by homogeneous morphological behaviour and affected by river dynamics in the last three decades (see section *ROIs definition*).

**Fig. 2:**
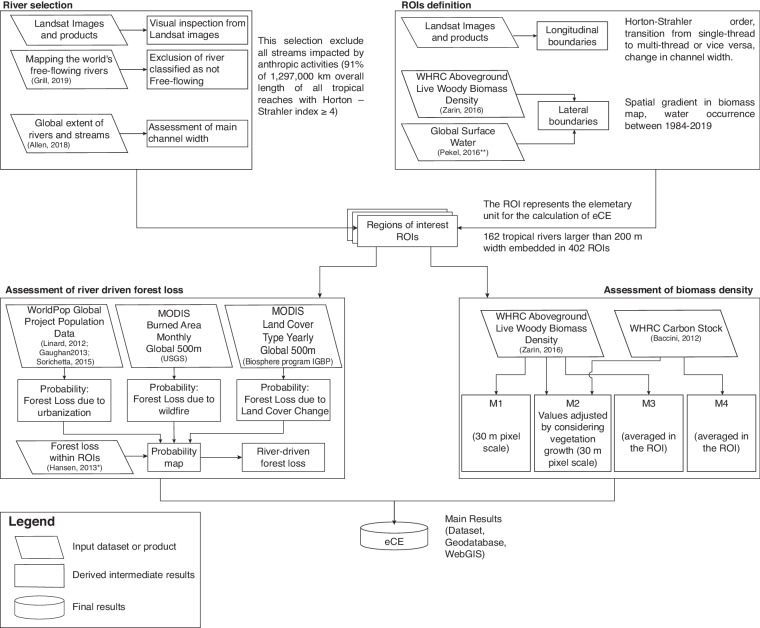
Flowchart of the framework used for the eCE analysis.

While the river selection procedure facilitated the identification of river segments with minimal anthropogenic influence on the interaction between rivers and terrestrial regions, it does not preclude the possibility that human-induced or non-river-related events may still contribute to forest loss. To ensure accurate quantification of carbon export attributable solely to River-Driven Forest Loss (RDFL), a probabilistic classification model was used to identify forest loss in the selected floodplain regions. This model – described in the *Probabilistic Classification Model* section – determines the probability P_*j, k*_ of RDFL occurrence in each pixel of the ROI by integrating the Global Forest Change dataset^26^ with three potential drivers of forest loss unrelated to rivers: (1) population density, (2) wildfires, and (3) land cover changes. For each pixel *k* of ROI *j* in which forest loss occurred, the model assessed the likelihood that the forest cover change was *not* due to urbanization (${{\rm{P}}}_{jk}^{(u)}$), wildfire (${{\rm{P}}}_{jk}^{(wf)}$), or anthropogenic land-cover changes (${{\rm{P}}}_{jk}^{(lc)}$), resulting in three different likelihood maps (see *Probabilistic classification model for forest change*). The overall likelihood map was obtained by multiplying the three probability maps, since they refer to independent events, namely ${{\rm{P}}}_{jk}={{\rm{P}}}_{jk}^{(u)}\cdot {{\rm{P}}}_{jk}^{(wf)}\cdot {{\rm{P}}}_{jk}^{(lc)}$. While there may exist a potential correlation between urbanization and alterations in land-cover changes, we have chosen not to incorporate these factors into our analysis due to their inherent challenges in large-scale quantification. Consequently, our assessment of forest loss driven by river dynamics tends to be more conservative. Extreme likelihood values are P_*j, k*_ = 0 (no forest loss or forest loss undoubtedly due to causes other than river dynamics), and P = 1 (forest loss undoubtedly due to river geomorphic activity). The eCE of the *j*-th ROI, expressed as TgC exported per year (in the form of woody biomass), was calculated as

1 $$eC{E}_{j}=\sum _{k}eC{E}_{j,k}=\sum _{k}{L}_{j,k}\cdot {\rho }_{j,k}$$

where *ρ*_*j, k*_ is the biomass density [TgC/km^2^] and *L*_*j, k*_ is the annual mean RDFL [km^2^ /year] for the period 2000–2019 and for pixel k of *ROI*_*j*_. To statistically exclude non-riverine causes, *L*_*j, k*_ was calculated as the product of the cell area *A*_*j, k*_ and the probability *P*_*j, k*_ that the loss is RDFL (see section “Probabilistic Classification Model”). We used four different methods to assess biomass density (M1-M4, see section *Biomass Density Assessment*). A graphical summary of the whole methodology is reported in Fig. 2.

### Global datasets used in the study as input

In the present work, several global databases were analyzed, as listed in Table 1, to investigate different aspects related to carbon dynamics and river activity. (1) The first dataset, WHRC Carbon Stock, provides a national-level map of aboveground live woody biomass density for tropical countries at a resolution of 500 m. It incorporates field measurements, LiDAR observations, and Moderate Resolution Imaging Spectroradiometer (MODIS) imagery^27^. This dataset was used to estimate carbon density within regions of interest, calibrate plant growth models (see *Biomass Density Assessment*), and classify the distribution signature of biomass (see *Definition of Carbon Signature*). (2) The WHRC Aboveground Live Woody Biomass Density dataset extends the methodology presented in ref. ^27^ and provides a global map of aboveground biomass at approximately 30 meter resolution^28^. It was used in this study to estimate carbon density within regions of interest and to calibrate plant growth models (see *Biomass Density Assessment*). (3) The Global Forest Change dataset provides information on global forest extent and change through time series analysis of Landsat imagery^26^. This dataset, specifically version 1.7 covering the period 2000–2019, was used to assess river-driven forest loss in the study (see *assessment of river-driven forest loss*). (4) The Global Surface Water dataset provides maps and statistics on the location, temporal distribution, extent, and changes of surface water from 1984 to 2019^29^. The dataset, specifically version 1.2 covering the period 2000–2019, has been used to assess wetlands within regions of interest (see *ROI definition*). (5) The MODIS Burned Area Monthly Global 500 m dataset provides per-pixel burned area and quality information at a monthly global gridded resolution of 500 m^30^. It was used to define the probability map of riverine forest loss (see *Riverine Forest Loss Assessment*). (6) The WorldPop Global Project Population Data provides high-resolution data on the distribution of human populations worldwide^31–33^. This dataset was used to define the probability map of riverine forest loss (see *Riverine Forest Loss Assessment*). (7) The MODIS Land Cover Type Yearly Global 500 m dataset provides global land cover types at yearly intervals from 2001 to 2019, derived from six different classification schemes^34^. It was used to define the probability map of river-driven forest loss (see *River-driven forest loss assessment*). (8) The Free Flowing Rivers dataset maps the world’s free-flowing rivers, identifying natural river reaches unaffected by human activities^23^. It was used to perform the identification of unimpacted river reaches (see *River selection*). (9) The HydroATLAS and RiverATLAS database^35^ provides a wide range of hydro-environmental attributes from existing global datasets in a consistent and organized manner. It was used for river selection and data filtering.

**Table 1:** List of the global databases used in the present work.

Dataset		Description	Data source	Use in this paper (Section)
1)	WHRC Carbon Stock^27^	A national-level map of above-ground live woody biomass density for tropical countries at 500 m resolution. This dataset was assembled from a combination of co-located field measurements, LiDAR observations, and imagery recorded from the Moderate Resolution Imaging Spectroradiometer (MODIS).	Baccini (2012)	Estimation of carbon density within regions of interest (eCE Computation - Method M4), Calibration of model for plant growth (SI-Logistic growth model – Method M2), (Classification of the biomass distribution signature).
2)	WHRC Aboveground Live Woody Biomass Density^28^	Global-scale, map of aboveground biomass (AGB) at approximately 30-meter resolution. This data product expands on the methodology presented in Baccini *et al*. (2012) to generate a global map of aboveground live woody biomass density (megagrams biomass ha-1) for the year 2000.	Zarin (2016)	Estimation of carbon density within regions of interest (eCE Computation – Methods M1, M2, M3), Calibration of model for plant growth (SI-Logistic growth model- Method M2).
3)	Global Forest Change^26^	Results from time-series analysis of Landsat images to characterize global forest extent and change.	Hansen (2013)* dataset version 1.7 (2000–2019)	Identification of river-driven forest loss RDFL (River selection and data filtering).
4)	Global Surface Water^29^	Maps of the location and temporal distribution of surface water from 1984 to 2019 and statistics on the extent and changes of those water surfaces.	Pekel (2016)** dataset version 1.2 (2000–2019)	Assessment of wet area within regions of interest (ROIs definition).
5)	MODIS Burned Area Monthly Global 500 m^30^	The Terra and Aqua combined MCD64A1 Version 6 Burned Area data product is a monthly, global gridded 500 m resolution product containing per-pixel burned-area and quality information.	USGS (2000-2019)	Definition of probability map of river-driven forest loss loss ${{\rm{P}}}_{j,k}^{(wf)}$ (River selection and data Filtering).
6)	WorldPop Global Project Population Data^31–33^	Global high-resolution, contemporary data on human population distributions.	Linard (2012) Gaughan (2013) Sorichetta (2015)	Definition of probability map of river-driven forest loss loss ${{\rm{P}}}_{j,k}^{(u)}$ (River selection and data Filtering).
7)	MODIS Land Cover Type Yearly Global 500 m^34^	The MCD12Q1 V6 product provides global land cover types at yearly intervals (2001–2019) derived from six different classification schemes.	Biosphere Programme classification (IGBP)	Definition of probability map of river-driven forest loss ${{\rm{P}}}_{j,k}^{(lc)}$ (River selection and data filtering).
8)	Free Flowing Rivers^23^	Mapping the world’s free-flowing rivers.	Grill (2019)	Identification of natural river reaches not impacted by human activities CSI (River selection and data filtering).
9a) 9b)	HydroATLAS RiverATLAS^35^	Comprehensive database presenting a wide range of hydro-environmental attributes from existing global datasets in a consistent and organized manner.	Linke (2019)	Assessment of Strahler index of river reaches (River selection and data filtering).

*Dataset updated annually, version 1.7 was used in this study which analyzes the period 2000–2019. **Dataset updated annually, version 1.3 was used in this study which analyzes the period 2000–2019.

### River selection

In order to ensure the strict adherence of eco-morphodynamic Carbon Export (eCE) quantification to the RDFL concept, a comprehensive two-step selection procedure was employed to identify and exclude cases that did not meet the RDFL criteria, particularly floodplain areas impacted by anthropic activities.

Step 1: **Identification of anthropogenic alterations through visual inspection of Landsat Imagery and analysis of Connectivity Status Index (CSI)**^23^

The first step involved a meticulous visual inspection of Landsat imagery to identify evident sources of anthropic alteration. Various physical infrastructures within the river channel and along the surrounding floodplain were scrutinized, including river channelization, check dams, weirs, fords, embankments, bank protection measures, revetments, and mining activities. Due to the presence of these alterations, all large Indian rivers were excluded from the analysis. This exclusion was necessary to ensure that the quantification of eCE focused exclusively on rivers that were minimally affected by significant human-induced modifications. Rivers were further classified based on the Connectivity Status Index (CSI) introduced by ref. ^23^. The CSI index serves as an indicator of fluvial connectivity and takes into account factors such as fragmentation, regulation, and alterations in water quality and temperature. Rivers classified as “not free-flowing” according to the CSI index (CSI index <95%) were excluded from the study. This criterion was applied to exclude rivers where fluvial connectivity was compromised due to anthropogenic factors.

Step 2: **Assessment of the main channel width**

The second step is to select only those rivers with a channel width exceeding 200 m when considering the mean annual discharge. River width is assessed according to ref. ^25^. This selection essentially depends on the spatial resolution of the available data sets. The satellite products^26^ currently available to study forest change during the last two decades at the global scale are based on the detection of stand disturbance or complete removal of tree canopy at the Landsat pixel scale (30 m). Similarly, the water surface detection datasets are based on Landsat imagery and can detect rivers greater than 30 m. However, the data are most accurate and complete at widths greater than 90 m^25^ (approximately three Landsat pixels). Assuming that also sinuous/meandering rivers can be locally characterized by at least a two-channel pattern, it was considered in this analysis only rivers with a main channel width greater than 6.5 Landsat pixels (i.e., 200 m).

The implementation of the first two steps resulted in the exclusion of a substantial portion (91%) of the total length of all tropical reaches with a Horton-Strahler index greater than or equal to 4, amounting to 1,297,000 km. By employing this selection procedure, the study aimed to maintain the eCE quantification focused on rivers where predominantly the floodplain is reworked only by river dynamics, thereby enabling a more accurate assessment of the RDFL within the analysis.

### Identification of region of interest

Each river under investigation underwent a partitioning process into Regions of Interest (ROIs), characterized by homogeneous morphological characteristics. These ROIs represent the fundamental units for determining (eCE) and are defined by both longitudinal and lateral boundaries. Geomorphological criteria^36^, such as variations in Horton-Strahler order^37^, sinuosity, transitions between single-thread and multi-thread channels, or abrupt changes in channel width^25^, were used as indicators to delineate the longitudinal divisions between successive ROIs.

The lateral extension of the ROIs encompasses the adjacent land influenced by the dynamics of the river and potential flooding, where the presence and distribution of vegetation are impacted. This active lateral region was identified through a two-step process.

**Fig. 3:**
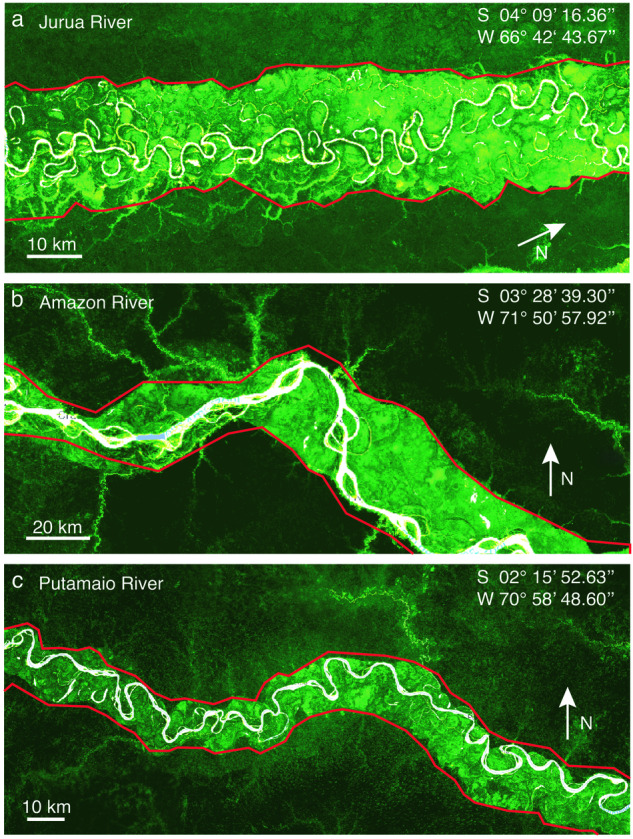
Three illustrative examples of carbon density maps derived from the dataset^28^ are presented, showcasing the lateral extension of Regions of Interest (ROIs) demarcated by red solid lines. These maps vividly emphasize the recurrent fluvial disturbances affecting floodplain vegetation, which in turn promote the continuous rejuvenation of riparian corridors. This process involves the removal of mature vegetation and subsequent colonization by seedlings and young trees along bare riverbanks. Consequently, it leads to an immature stage with diminished carbon stocks compared to non-flooded mature forests^38,56^. The discernible transition in carbon distribution between the disturbed floodplains and the adjacent terra firma is clearly depicted in the carbon map^28^. This distinct edge serves as the basis for defining the lateral boundaries of the ROIs, demarcated by the red lines. The specific locations shown in the figure are: (**a**) Jurua River; (**b**) Amazon river; and Putamaio River.

Firstly, the spatial gradient in biomass density was taken into account. Areas frequently affected by floods or river dynamics are characterized by vegetation that has adapted to withstand these conditions, resulting in a successional pattern with specific biomass distributions^38^. Using a high-resolution biomass map, we were able to identify the boundaries between floodplain forests and upland forests, commonly referred to as “tierra-ferma” in Amazonian basins (Fig. 3).

Secondly, in instances where delineating lateral boundaries from the biomass map proved challenging, we integrated information regarding water surface occurrences from the Global Surface Water (GSW) dataset^29^ (Table 1, dataset number 4). Through this approach, we were able to consistently identify regions that exhibited a consistent presence of water in the GSW dataset over the past 35 years, enabling the precise delineation of transitional zones between aquatic and terrestrial environments.

It is crucial to recognize that the GSW dataset may not provide precise detection of short-lived events due to the requirement of synchronizing observations with cloud-free satellite coverage. The tropical region, particularly the eastern Amazon Basin, is heavily affected by extensive cloud cover, which results in the omission of many intense but short-lived events from the event map developed by ref. ^29^. As a consequence, our estimations of the lateral boundaries of the ROIs tend to be more conservative.

### Probabilistic classification model for River-Driven Forest Loss assessment

A probabilistic classification model was employed in this study to determine the probability of RDFL occurrence for each pixel within the ROIs. To achieve this, the Global Forest Change dataset^26^ was filtered, taking into account three potential causes of non-RDFL: i) population density, ii) forest fires, and iii) land-cover changes (datasets n.5–7 of Table 1). Consequently, for each j-th pixel within the k-th ROI where forest loss was observed, the model evaluated the likelihood that the change in forest cover was not due to urbanization (${{\rm{P}}}_{jk}^{(u)}$), wildfire (${{\rm{P}}}_{jk}^{(wf)}$), or man-made land-cover changes (${{\rm{P}}}_{jk}^{(lc)}$), generating three probability maps. For the first map (${{\rm{P}}}_{jk}^{(u)}$), the model uses the spatial distribution of Population density as a proxy of the probability that the forest loss was caused by Urbanization, while ${{\rm{P}}}_{jk}^{(wf)}$ and ${{\rm{P}}}_{jk}^{(lc)}$ are based, in each pixel, on the temporal distance between the non-river driven event and the year of forest loss.

#### Forest loss due to urbanization

The values represented in the map ${{\rm{P}}}_{jk}^{(u)}$ decrease as the population density PD increases. Based on the relationship between human pressure scores and population density in sparsely populated areas suggested by ref. ^39^, we implemented the following equation:

2 $${P}_{j,k}^{({\rm{u}})}=\left(\begin{array}{c}\begin{array}{ll}1-0.333\cdot \log ({\rm{PD}}+1), & {\rm{for}}\;{\rm{PD}} < 1,000\quad {\rm{people}}/{{\rm{km}}}^{2}\\ 0 & {\rm{for}}\;{\rm{PD}}\ge 1,000\quad {\rm{people}}/{{\rm{km}}}^{2}\end{array}\end{array}\right.$$

The PD data was obtained from the WorldPop Project Population dataset^31–33,40^ at a resolution of 100 m (n.6 of Table 1).

#### Forest loss due to forest wildfire or land cover change

**Fig. 4:**
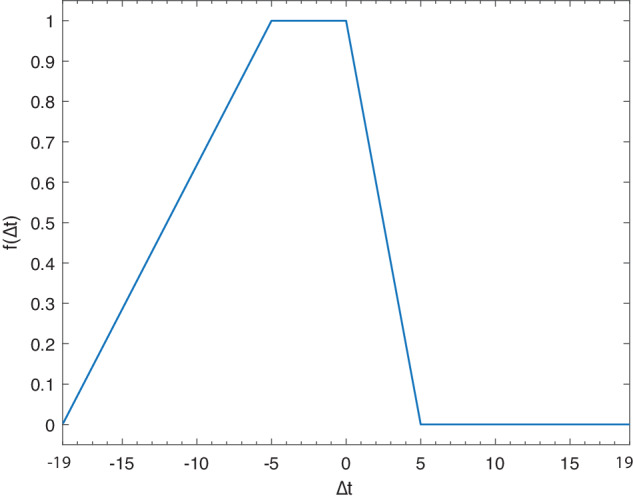
The function f (Δt) Probabilistic classification model for River-Driven Forest Loss assessment.

To define the maps ${{\rm{P}}}_{jk}^{(wf)}$ and ${{\rm{P}}}_{jk}^{(lc)}$, the probability that the forest loss in a given year has been caused by a non-River-Driven Event (henceforth referred non-RDE) was expressed as a function f(Δt), where Δt is the time gap (causal relation principle) between the forest loss and non-RDE occurred in the same region (wildfires or land cover changes). The function f(Δt) – i.e., the probability that the loss has been caused by a non-RDE – follows a piecewise dependence on time, as reported in Fig. 4.

Essentially, if the forest loss and the non-RDE belong to the same year (i.e., Δt = 0), the causal connection is guaranteed, so the function takes the maximum (f = 1). Cases with Δt < 0 imply that the non-RDE anticipated a forest loss. In these cases, a positive causal connection may be possible for several reasons. For example: i) the non-RDE might have not caused a detectable forest loss in the same year, e.g., a wildfire that irreversibly damaged the vegetation which however died in the following months/years; ii) extreme cloudiness of tropical region caused a delay in the forest loss detection. In the cases with Δt > 0, forest loss anticipated the non-RDE. Albeit counter-intuitive, even in these cases, a positive causal connection can be possible. For example, a slow land conversion (e.g., from forest to cropland) that takes some years to cover a portion of territory observable through a MODIS-based dataset (coarse resolution 500 m) while was suddenly detected as forest change in the Landsat-based products (resolution of 30 m). In each plot performing a forest loss during the observation window, fire events were detected by using the MODIS-based dataset^30^. We set

3 $${P}_{j,k}^{({\rm{wf}})}=\mathop{\prod }\limits_{i=1}^{N}1-{f}_{i}(\Delta t),$$

where N is the number of fires observed during 2000–2019 in the pixel. Where no fires were observed, ${{\rm{P}}}_{jk}^{(wf)}=1$. We remark that this filter excludes the capture of recalcitrant LWD generated by the incomplete combustion of biomass during fires, so-called black carbon as analyzed in^41^. This aspect may be an additional source of underestimation of the present eCE assessment. The map ${{\rm{P}}}_{jk}^{(lc)}$, namely the likelihood that forest loss is not due to land cover change caused by human activity, is generated by using the dataset n.7 in Table 1, MODIS Land Cover Type MCD12Q1^34^. Following the classification of the Annual International Geosphere-Biosphere Programme (IGBP, Table 1), four land cover macro-classes were identified: Natural

With High vegetation density (NHV), Natural with Low vegetation density (NLV), Anthropic (AN) and Water/Unvegetated (UV). NHV class comprise the areas classified as Land Cover – Type 1, as evergreen or deciduous needleleaf forest, evergreen or deciduous broadleaf forest, Mixed Forest, closed or open Shrublands, grasslands and permanent wetlands; NLV comprises savannas and woody savannas; AN class contains croplands, cropland or natural vegetation mosaics, urban and built-up land; UV encompasses water bodies, permanent snow and ice and barren. A per-pixel analysis at MODIS scale was performed in ROIs and each yearly variation in land cover macro class was detected and classified. In each pixel, the variations from NHV to NLV, from NHV to AN and from NLV to AN were considered due to human activities while all the other changes were attributed to river morphodynamic processes (i.e., RDFL).

**Fig. 5:**
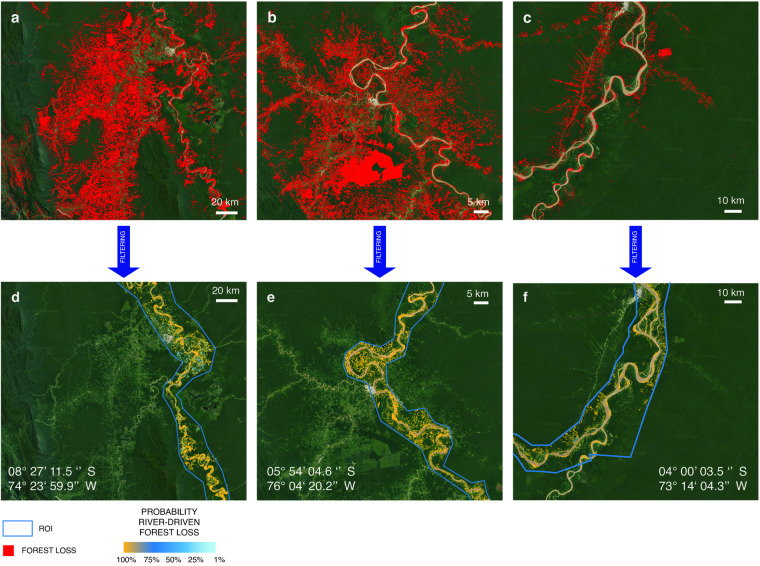
Filtering procedure. (**a**–**c**) Satellite images before filtering, with forest loss reported in red. (**d**–**f**) The same images after filtering, with the probability of River-Driven Forest Loss clustered in four classes (see legend).

The probability that the forest loss at pixel k of ROI j was not due to human–induced land cover change is therefore defined according to the same equation as Eq. (2) where N is intended as the number of land cover transitions observed during the 2000–2019 in the same pixel, while Δt is intended as the time difference between the forest loss and the land cover change. When no human-induced land cover variations were detected, ${{\rm{P}}}_{jk}^{(lc)}=1$. For the above reasons, a conservative choice in terms of eCE estimation was to assume that when forest loss and non-RDE occurred within the temporal window of five years they were causally connected, so f = 1. The result of the filtering procedure for three example cases is shown in Fig. 5.

### Assessment of biomass density

Defining the biomass content of areas that have experienced vegetation loss due to river dynamics is a key aspect of estimating eCE. Estimating the eCE requires knowledge of the carbon density value of the vegetation characterizing the j-th pixel undergoing RDFL at the time of the RDE. Although several biomass density satellite products have been made in recent decades, there are currently no globally available annual time–resolved data with a spatial resolution suitable for studying this process. For this reason, a comparison of 4 different indirect approaches based on two different robust biomass maps has been carried out.

Method M1: *ρ*_*j, k*_ was taken from the WHRC Carbon Stock dataset developed by^28^ for the above-ground living woody biomass density at 30 m resolution for the year 2000 (n.2 of Table 1). In this case, the carbon density of a single cell was assumed constant during the entire period of analysis, neglecting the possibility that plots, where the loss occurred after the year 2000, might have experienced an increase in the carbon content due to growth in the time between 2000 and the year of loss.

Method M2: The value of carbon density of each pixel was adjusted considering the amount of vegetation that had grown between the year 2000 and the year of loss, by using a calibrated logistic growth model (see next section).

Methods M3 and M4: The value of carbon density of each pixel was approximated using the spatial average over the whole ROI (i.e., ${\rho }_{j,k}={\sum }_{k}{\rho }_{j,k}/{N}_{j}$, being *N*_*j*_ the number of pixels in ROI j) by using the WHRC *Carbon Stock* datasets by ref. ^28^ for M3 and ref. ^27^ for M4 (datasets n.2 and n.1 of Table 1, respectively). These datasets describe biomass in tropical regions for only a limited period (the year 2000 for n.2 and the period 2007–2008 for n.1).

Tropical rivers are highly dynamic systems that during an inter-decade evolution likely visit most of their geomorphological configurations (e.g., the Ucayali River, a tributary of the Amazon River, shows migration rates of up to 100 m/year). For methods M3 and M4, we, therefore, adopted an ergodic-like hypothesis^42^, which allowed the temporal mean of carbon density in a single plot to be inferred from its spatial average over the whole ROI. It is worth noting that spatial averaging in methods M3 and M4 induces a slight underestimation of the eCE (see Table 5), since the erosion mechanism and the consequent capture of biomass usually involve the mature bank, where vegetation is at a higher level of growth.

Since the considered datasets only report the above-ground biomass (AGB) density, the belowground biomass (BGB) was assessed as BGB = 0.489·AGB^0.89^ (ref. ^43^), and the total carbon was estimated as 50% of the total biomass (AGB + BGB). We remark that the relative differences of the eCE estimation among methods M1-M4 does not exceed 3.3% (Table 5). Quantitatively, the four different methods, therefore, perform in a very similar way, despite they are based on different datasets. For simplicity, the results reported in the main text refer to Method 2.

#### Calibration of the logistic growth model update in Method M2

The method M2 considers the increase in the carbon content due to vegetation growth between the dataset acquisition time (year 2000, ref. ^28^) and the time of forest loss. According to the approach proposed by ref. ^44^, the increase in carbon content was addressed through the calibration of a simplified logistic biomass growth model^45,46^,

4 $$\frac{d{\rho }_{i}}{dt}={\alpha }_{i}{\rho }_{i}\left({V}_{i}-{\rho }_{I}\right)$$

where *ρ* represents the biomass carbon density, *t* is time, *V* stands for the carrying capacity, which is the maximum sustainable biomass carbon density, *α* indicates the growth rate specific to each vegetation species while subscript the *i* refers to the generic *i*-th cell. Considering the initial values of biomass carbon density the ones reported by the dataset^28^ at year *t*_0_ = 2000 (i.e. *ρ*_0, *i*_ = *ρ*(*t*_0_)) the formal solution of Eq. (6) at time *t* = *t*_0_ + Δ*t*, for a generic species community reads

5 $${\rho }_{i}=\frac{{A}_{i}{\rho }_{0,i}{V}_{i}}{\left({A}_{i}-1\right){\rho }_{0,i}+{V}_{i}}$$

where we have defined ${A}_{i}=\exp ({V}_{i}{\alpha }_{i}\Delta t)$. The function A_*i*_ and the parameter V_*i*_ were locally calibrated through the following procedure. That allows us to use the Eq. (7) to update the value of carbon biomass density from *t* = *t*_0_ = 2000 to the time of the cover loss ($t={t}_{0}+\Delta t$), in any cell. The calibration process is based on evaluating the carbon biomass data from two distinct datasets collected eight years apart (ref. ^28^, and ref. ^27^ corresponding to years 2000 and 2008 respectively, labeled as n.2 and n.1 in Table 1). These datasets are comparable because they were produced using the same methodology, albeit at different resolutions (30 meters per pixel for ref. ^28^, and 500 meters per pixel for ref. ^27^). The calibration procedure relies on the comparison of carbon biomass as reported by two different datasets with acquisition times eight years apart (ref. ^28^ and ref. ^27^ referring to 2000 and 2008, respectively n.2 and n.1 in Table 1). The comparison of these two datasets is possible since they were generated by the same methodology, albeit with different resolutions (30 mpx for ref. ^28^ and 500 mpx for ref. ^27^). In the following, the two datasets will be tagged with subscripts _30_ and _500_, respectively. Firstly, all cells in the 30 m resolution dataset were resampled to the 500 m resolution within blocks corresponding to the pixel boundaries of the second dataset. Secondly, for each j-th block, we imposed the matching between the mean of the values *ρ*_30, *i*_ within the block (updated at t = 2008) and the value *ρ*_30, *i*_, namely,

6 $$\frac{1}{{N}_{j}}\mathop{\sum }\limits_{i=1}^{{N}_{j}}{\left[{\rho }_{30,i}\right]}_{t=2008}={\rho }_{500,j}$$

which, after using Eq. 5, becomes

7 $$\frac{1}{{N}_{j}}\mathop{\sum }\limits_{i=1}^{{N}_{j}}\frac{{A}_{i}{\rho }_{0,i}{V}_{i}}{\left({A}_{i}-1\right){\rho }_{0,i}+{V}_{i}}{| }_{\Delta t=8years}={\rho }_{500,j},$$

where N_*j*_ is the number of 30 m resolution cells in the j-th 500 m resolution block. Thirdly, the assumption was made that all cells within every block possess identical values for A*i* and V, thus A*i* = A is a constant that may be extracted from the summation in Eq. (9). Additionally, given that 1/*ρ*0, *i* (A-1) $\approx 1/\rho 0,i\gg 1/{\rm{V}}$, as a first-order approximation, we obtain

8 $$A{| }_{\Delta {t}^{* }}\approx \frac{{\rho }_{500,j}}{{\rho }_{M}},$$

where ${\rho }_{M}={N}_{j}^{-1}{\sum }_{i=1}^{Nj}{\rho }_{0,i}$. Through iterative substitution of Eq. 8 into Eq. 7, a second-order approximation is attained:

9 $$A{| }_{{\Delta }^{* }}\approx \frac{{N}_{j}{\rho }_{500,j}}{\mathop{\sum }\limits_{1}^{{N}_{j}}\frac{1}{\frac{\frac{{\rho }_{500,j}}{{\rho }_{M}}-1}{V}+\frac{1}{{\rho }_{i}}}}$$

Recursive analysis shows that additional approximations result in a cumbersome formula with a continued fraction in the denominator of Eq. 9. For practical computational purposes, it is adequate to terminate the process at the second step. The carrying capacity was cautiously assumed constant throughout the ROI and equal to the maximum value of *ρ*_*M*_ (namely, ${\rm{V}}={\rho }_{M}^{max}$). By replacing in Eq. 5, and after recalling that, by definition,

10 $$A| \Delta t={(A{| }_{\Delta {t}^{* }})}^{\frac{t}{\Delta {t}^{* }}}$$

where Δt^*^ = 8 yr is the time lag between the two datasets, one finally gets the relationship for the carbon density updated at time t, for each cell:

11 $${\rho }_{i}(t)=\frac{\left(A{| }_{\Delta {t}^{* }}\right){\rho }_{0,i}{\rho }_{M}^{max}}{[{\left(A{| }_{\Delta {t}^{* }}\right)}^{\frac{t}{\Delta {t}^{* }}}-1]{\rho }_{0,i}+{\rho }_{M}^{max}}$$

### Carbon signature and classification algorithm

According to ref. ^7^, the ecological and morphodynamic carbon export leaves a distinctive signature on biomass distribution. This is influenced by downstream variations in waterlogging duration (hydroperiod) and the fluvial platforms.

In single-thread meandering rivers with high migration rates, mature forests are eroded laterally, while sediment deposition provides fertile ground for young vegetation to establish. The short hydroperiod allows the forest to reach maturity and store large amounts of carbon. Conversely, point bars and bare banks are rapidly colonized by seedlings and young trees with high carbon sequestration potential but lower carbon density, resulting in a negatively-skewed carbon distribution. In multi-thread rivers, where fluvial disturbances are more pronounced, islands and banks experience varying vegetation dynamics. Under weakly disturbed conditions with a short hydroperiod, mature forests populate islands or central bars, while young trees develop along the banks, yielding a multi-modal carbon density distribution. With increasing Horton-Strahler order, the hydroperiod typically lengthens, inhibiting the development of mature vegetation in island cores, and maintaining the system in a juvenile state, resulting in a positively-skewed carbon density distribution.

**Fig. 6:**
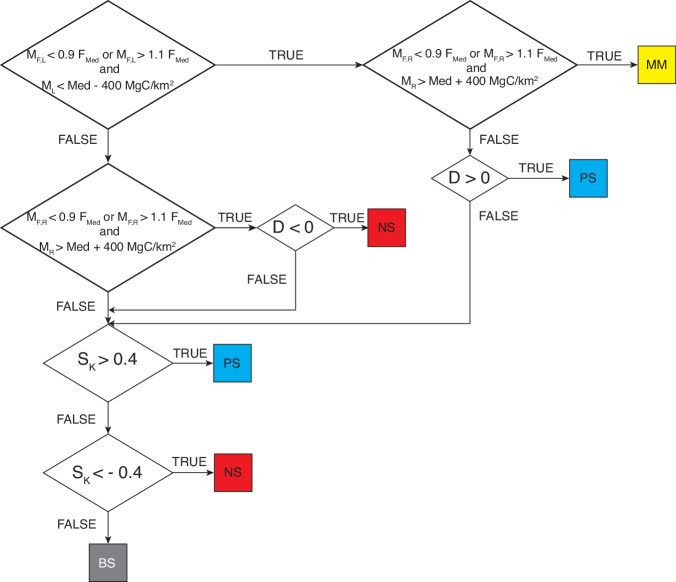
Carbon signature classification algorithm. The algorithm relies on comparing the statistical parameters within each ROI of carbon density distribution (specifically frequency of median value F_*med*_, median value Med, skewness S_*k*_) with the statistical parameters of two sub-samples extracted. These sub-samples are obtained by dividing the data of each ROI, using a cutoff set at the median value of the carbon density distribution and are referred to as the left- (L) and right- (R) sub-samples. The statistical parameters of sub-samples used for the analysis are the frequency of the modes (F_*ML*_ and F_*MR*_), the modes (M_*L*_ and M_*R*_), the difference D = F_*MR*_ – F_*ML*_. Further details about the algorithm are provided by ref. ^7^.

**Fig. 7:**
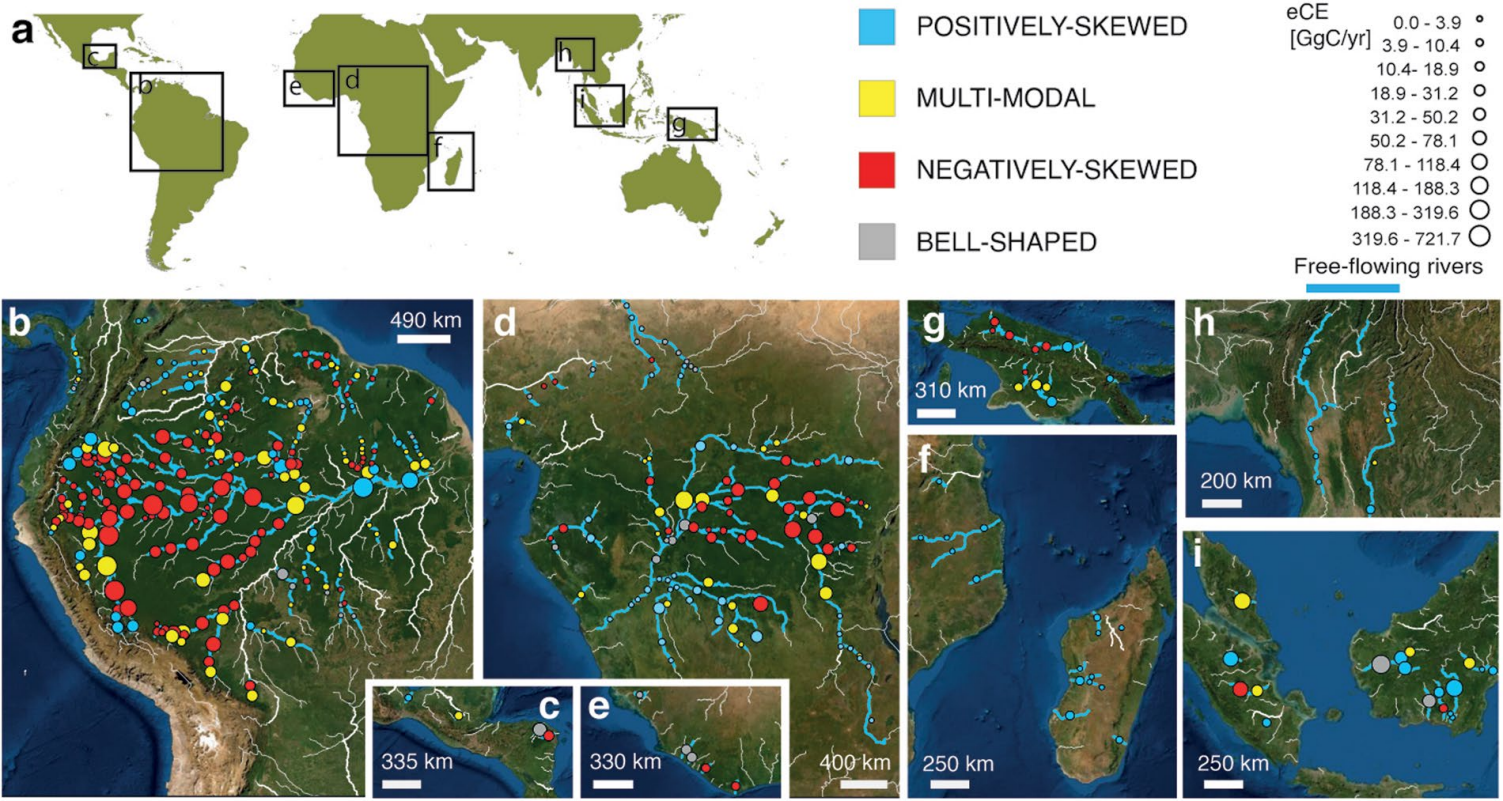
Carbon Signatures. Maps of the carbon signature of the world’s largest free-flowing tropical rivers in America (**a**,**b**), Africa (**c**–**e**) and Asia (**f**–**h**).

Through the examination of the WHRC Carbon Stock dataset^27^ and the application of the clustering algorithm (Fig. 6) proposed by ref. ^7^, four distinct signatures of fluvial biomorphological activity in biomass distribution were identified within ROIs: negatively-skewed (NS), positively-skewed (PS), multimodal (MM), and bell-shaped (BS). In the present study, 392 ROIs were analyzed (10 ROIs were excluded because they fell in areas not covered by WHRC Carbon Stock dataset^27^). The results are shown in the Fig. 7m. Moreover, the results aggregated at catchment scale are summarized in Table 2.

**Table 2:** Results of the classification algorithm and partition of the carbon signature at continental scale.

	NS	MM	PS	BS
**Catchment scale**				
**America**				
Upstream Amazon Basin	66.7%	22.2%	11.1%	0%
Central Amazon Basin	60.0%	30.7%	5.3%	4.0%
Downstream Amazon Basin	27.4%	45.4%	15.1%	12.1%
Others	18.5%	29.6%	38.9%	13.0%
**Africa**				
Upstream Congo Basin	54.3%	28.3%	8.7%	8.7%
Downstream Congo Basin	4.2%	20.8%	70.8%	4.2%
Others	15.9%	6.8%	47.7%	29.6%
Madagascar	0.0%	0.0%	100.0%	0.0%
**Asia**				
New Guinea Island	50.0%	25.0%	25.0%	0.0%
Borneo Island	7.7%	15.4%	61.5%	15.4%
Sumatra Island	25.0%	25.0%	50.0%	0.0%
Malay Peninsula	0%	100.0%	0.0%	0.0%
Others	0%	50.0%	50.0%	0.0%
**Continental scale**				
America	47.9%	29.9%	16.2%	6.0%
Africa	26.7%	16.9%	41.9%	14.5%
Asia	23.5%	26.5%	44.1%	5.9%
**Global scale**				
Tropics	39.0%	25.5%	26.8%	8.7%

NS: negatively skewed; MM: multi-modal; PS: positively skewed; BS: bell-shaped.

**Fig. 8:**
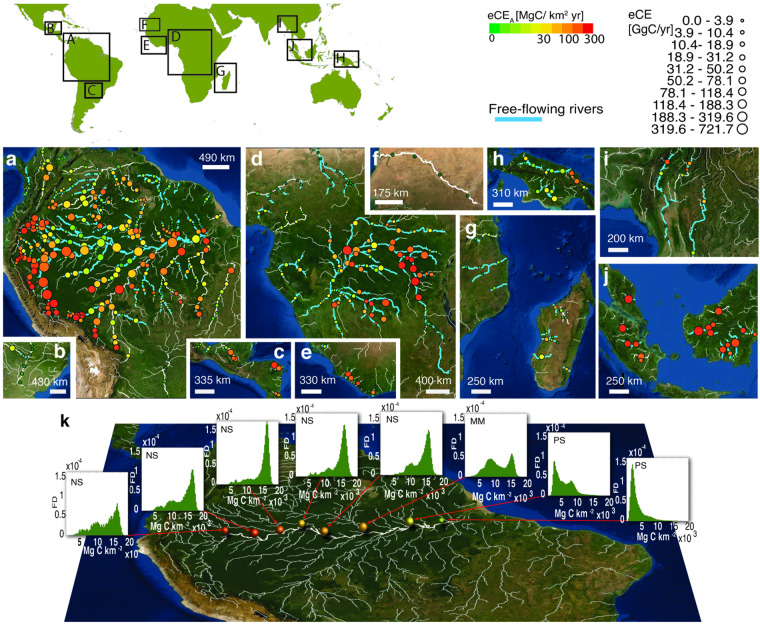
Eco-morphodynamic Carbon Export (eCE) of the world’s largest tropical rivers (overall 402 ROIs). (**a**) South America, (**b**) Northern Argentina, (**c**) Mexico, (**d**) Central Africa, (**e**) Central West Africa, (**f**) West Africa, (**g**) Southeast Africa, (**h**) Papua and New Guinea, (**i**) Southeast Asia, (**j**) Borneo and Sumatra. Point size is proportional to eCE, colors show eCE_*A*_. Blue reaches indicate free-flowing streams (CSI index > 95%, after ref. ^23^). (**k**) Longitudinal sequence of signatures in the frequency distribution for Amazon River corridor biomass density (NS: negatively skewed; MM: multimodal; PS: positively skewed.).

This study confirms, as observed by ref. ^7^, that fluvial corridors generally follow an NS-MM-PS pattern as the Horton-Strahler number increases^37^, as is evident in the river analyzed (Fig. 8). These signatures serve as proxies for river export capacity and highlight the interplay between sediment transport, flood dynamics, river morphology, and carbon transport Table 3.

**Table 3:** Estimates of Eco-morphodynamic Carbon Export (eCE) and River-Driven Forest Loss Area (A_*RDFL*_) for the largest tropical rivers.

Basins	eCE_*A*_ [MgC/km^2^yr]	eCE [TgC/yr]	A_*RDFL*_ [km^2^/yr]
**America**			
Upstream Amazon	63.6	4.28(34.4%)	295
Central Amazon	24.7	3.13 (25.2%)	212
Downstream Amazon	19.4	1.08 (8.7%)	94
Others	17.2	0.41 (3.3%)	36
**Africa**			
Upstream Congo	53.7	1.65 (13.3%)	118
Downstream Congo	31.8	0.34 (2.8%)	30
Others	10.6	0.18 (1.4%)	21
Madagascar	14.4	0.06 (0.5%)	9
**Asia**			
New Guinea Island	12.9	0.18 (1.4%)	14
Borneo Island	71.3	0.7 (5.6%)	61
Sumatra Island	100.3	0.22 (1.8%)	21
Malay Peninsula	149.8	0.13 (1.0%)	11
Others	20.3	0.09 (0.7%)	8
**Major Exporters**			
**River**	**eCE [Tg C/yr]**	**Rivers**	**eCE [Tg C/yr]**
Amazon	2.6 (21.2%)	Congo	0.37 (2.9%)
Ucayali	1.4 (11.5%)	Ubangi	0.32 (2.5%)
Rio Negro	0.42 (3.4%)	Lualaba	0.31 (2.5%)
Purus	0.38 (3.1%)	Maranon	0.31 (2.5%)
Kapuas	0.37 (3.0%)		

Values in parentheses indicate the percentage relative to total eCE = 12.45 Tg C/yr. Uncertainty analysis is described in Methods.

## Data Records

The results of the analysis, summarized in the Figs. 7, 8, are stored in an online figshare repository (10.6084/m9.figshare.24794295.v2^20^ as a stand-alone table, an ESRI GeoDatabase (created to enable the implementation of a WebGIS application) and a GeoPackage (an open, standards-based, platform-independent, portable, self-describing, compact format for transferring geospatial information, OGC).

The repository follows the FAIR principle of Findability, Accessibility, Interoperability and Reusability of data. Specifically, starting from the standalone table and the geometries of the ROI polygons (referenced to the WGS84 datum in geographical coordinates, EPSG:4326), an ESRI file geodatabase (see ESRI website for definitions) was created and populated with two feature classes with the same attributes.

A polygon feature class related to the ROIs and a point feature class related to the centroids of each ROI polygon (used at smaller map scales in the WebGIS environment, to ensure map readability). The attribute table of the ROI feature class contains 22 fields and 402 records. More in detail, the attribute table provides for each polygon (i.e. ROI) the main results of the study (eCE, eCEA, carbon signature), general information of the ROI (name and identification code of the river of which the ROI is part, code with which to identify other ROI, coordinates of the polygon that define the ROI in GeoJSON format), and the fundamental variable for the analysis (Area of river driven forest loss, ROI Area, Carbon Signature). A synthetic description of all available data is provided in Table 4. The dataset described above is released under the Creative Commons Attribution licence (CC-BY, v. 4.0, Creative Commons licenses).

**Table 4:** List and description of all the data fields present in the geodatabase.

Data Field	Field Definition	Field Type	Unit
A_*ROI*_	ROI Area	Number - Double	km^2^
A_*RDFL*_	River-driven forest loss area	Number - Double	km^2^/yr
Basin code	Basin or region identification code	Number - Integer	—
Basin or region Name	Name of the basin or region to which the ROI belongs	Text	—
Carbon signature code	Numerical code to identify carbon signature. 1 = Negatively Skewed, 2 = Multi-Modal, 3 = Positively Skewed, 4 = Bell-shaped	Number - Integer	—
Carbon signature description	Carbon Signature classes	Text	—
code	River identification code	Text	—
eCE_*M*1_	Eco-morphodynamic carbon export (Method carbon density assessment M1)	Number - Double	TgC/yr
eCE_*M*2_	Eco-morphodynamic carbon export (Method carbon density assessment M2)	Number - Double	TgC/yr
eCE_*M*3_	Eco-morphodynamic carbon export (Method carbon density assessment M3)	Number - Double	TgC/yr
eCE_*M*4_	Eco-morphodynamic carbon export (Method carbon density assessment M4)	Number - Double	TgC/yr
eCE_*AM*1_	Eco-morphodynamic carbon export per ROI unit area (Method carbon density assessment M1)	Number - Double	MgC/km yr
eCE_*AM*2_	Eco-morphodynamic carbon export per ROI unit area (Method carbon density assessment M2)	Number - Double	MgC/km^2^ yr
eCE_*AM*3_	Eco-morphodynamic carbon export per ROI unit area (Method carbon density assessment M3)	Number - Double	MgC/km^2^ yr
eCE_*AM*4_	Eco-morphodynamic carbon export per ROI unit area (Method carbon density assessment M4)	Number - Double	MgC/km^2^ yr
FFR	Free-flowing classification based on ref. ^23^	Integer (0 or 1 value)	—
geo	Geometric coordinates of the ROI, GeoJSON format. WGS84 datum in geographical coordinates, EPSG:4326	Text	—
Name	River name	Text	—
roi code	Identification code of the ROI within the river reach	Text	—
State	State in which the ROI is contained (for ROIs that border multiple states, the state in which the largest area fraction of the ROI falls)	Text	—
unicode	Unique ROI identification code (*code* concatenated with *roi code*)	Text	—
URL	Link to Carbon density distribution figure	Text	—

The geodatabase comprises:

- 402 river reaches, classified as free-flowing according to ref. ^23^, embedded in ROIs. The attribute field *basin* identifies, through a numerical code, 13 regions or sub-regions considered geomorphologically homogeneous (Table 3).
  - The Amazon basin and corresponding tributaries can be divided into three geomorphologically homogeneous sub-regions. The upstream region (identified with the basin code equal to 1), corresponding to the Peruvian-Bolivian Amazon basin, is the most dynamic one (eCE = 4,283 GgC/yr, see Table 3) with high levels of sinuosity, bank erosion rate and channel migration. The Lowland Rainforests in such a region are heavily influenced by lateral erosion of meandering rivers and new sequential succession forest develops on scroll bars very rapidly, while most of the (mature) mosaic vegetation loss is on the outer bank or in the short-lived islands^47^.
  - The middle region (basin code 2) is characterized by a lower erosion rate and more stable channel banks (eCE = 3,132 GgC/yr, see Table 3). Meandering rivers (e.g., such as Purus, Jurua, Jutai) have migration rates lower than 0.2 channel-widths/yr^48^ (because of the low levels of sediment transport) and an eCE_*A*_ between 1.4 and 100 MgC/km^2^/ yr. The Amazon River corridor of this region is characterized by an increase in the recurrence of low-waters, and green grass and shrubs species colonize a rising portion of wetlands with the consequent reduction of woody plant communities^49^. For instance, the Negro river corridor (a tributary of the Amazon River in Central Amazonia) is characterized by relatively lower biomass density where swamp forest (igápo) and white sand vegetation populate stable islands^50^.
  - The downstream subregion (e.g. Jurunea, Rio Mapuera, basin code equal to 3) provides the lowest levels of eCE in the Amazon basin (eCE = 1,079 GgC/yr see Table 3, eCE_*A*_ = 19.4 MgC/km^2^ yr). The Amazon River corridor in this region is populated by dish-shape lakes in the floodplain and herbaceous vegetation is widespread. Carbon pumping is dominated by recurrent floods, so vegetation remains at the juvenile stage and biomass density is usually low ( < 53 MgC/ha). However, the amount of carbon sequestrated remains high due to a high river-land connectivity (800 GgC/yr) while eCE_*A*_ is lower than the upstream zone. Other rivers outside the Amazon Basin (Orinoco basin) and rivers of Central America, identified for simplicity with a single basin code (4) even though they actually belong to different basins, exported 407 GgC/yr with eCE_*A*_ that ranges between 1 and 102 MgC/km^2^ yr (mean value: 17 MgC/km^2^).
  - African eCE is mainly due to the Congo basin, which can be divided in two subregions. The upper region (the Upper Congo River and Lowa River), identified with basin code 5, is characterized by dense forests with high values of above-ground biomass (120 MgC/ha). The vegetation is here mainly removed by overflow and uprooting, the planforms are stable and long-lived and vegetation populates both the floodplain and the riparian corridor. In contrast, the central-downstream region of the Congo basin (identified with basin code 6) represents one of the world’s most extensive swamp forests, which is supposed to host a huge peat deposit (30.6 PgC, ref. ^51^). Nevertheless, the density of above-ground biomass is modest (66 MgC/ha) and the mean sequestration capacity is 31 MgC/km^2^ yr. The remaining African basins, identified for simplicity with a single basin code (7), export only 177 GgC/yr^−1^ (Table 3). This is due to a low tree cover and aboveground carbon density (39 MgC/ha) that characterize arid and semiarid zones.
  - The Indonesian and New Guinea forest has a relevant carbon store 18.6 PgC, ref. ^27^, but it experimented with an important conversion from tropical forest to oil palm plantation^52^. Also, the historically unaltered wetland forest has lost ~ 52,000 km^2^ of cover between 2000 and 2012^53^, probably due to agro-industrial land development^53^. These aspects are not related to fluvial dynamics, and make the assessment of river-induced forest loss more uncertain (see Table 5). Our assessment of carbon exported by Indonesian basins (basin code 9–13) is 1,840 GgC/yr (Table 3) and eCE_*A*_ ranges between 2.9 and 149.8 MgC/km^2^yr (mean value: 54.4 MgC/km^2^yr).

**Table 5:** Results about the aggregated continental eCE (by using the methods M1-M4) and the corresponding uncertainties, for the largest tropical free flowing rivers (width > 200 m.).

Methods	Continental eCE (TgC/yr)			Autocorrelation scale	Carbon uncertainties	Continental eCE uncertainties (TgC/yr)		
	America	Africa	Asia			America	Africa	Asia
M1	8.60	2.20	1.17	500 m	50% *ρ*_*j, k*_	0.06	0.03	0.02
					75% *ρ*_*j, k*_	0.09	0.04	0.03
					100% *ρ*_*j, k*_	0.12	0.06	0.05
					125% *ρ*_*j, k*_	0.15	0.07	0.06
				ROI scale	50% *ρ*_*j, k*_	0.39	0.17	0.13
					75% *ρ*_*j, k*_	0.51	0.22	0.18
					100% *ρ*_*j, k*_	0.67	0.29	0.23
					125% *ρ*_*j, k*_	0.84	0.36	0.29
M2	8.89	2.23	1.25	500 m	50% *ρ*_*j, k*_	0.06	0.03	0.02
					75% *ρ*_*j, k*_	0.09	0.04	0.03
					100% *ρ*_*j, k*_	0.12	0.06	0.05
					125% *ρ*_*j, k*_	0.15	0.07	0.06
				ROI scale	50% *ρ*_*j, k*_	0.39	0.17	0.13
					75% *ρ*_*j, k*_	0.51	0.22	0.18
					100% *ρ*_*j, k*_	0.67	0.29	0.23
					125% *ρ*_*j, k*_	0.84	0.36	0.29
M3	7.91	2.01	1.02	ROI scale	Spatial St.Dev. *ρ*	0.22	0.09	0.06
M4	8.62	2.11	1.01	ROI scale	Spatial St.Dev. *ρ*	0.23	0.09	0.06
  - The dataset contains the result of carbon signature classification analysis for each ROI analysed. Overall, it was observed that 47.9% of observations are NS, 29.9% are PS, 16.2% are MM and 6.0% are BS. The results of a statistic analysis at the basin scale are reported in Table 2.
- A further set of 115 ROIs, selecting from the rivers classified by ref. ^23^ as not free-flowing, in order to investigate the extent to which river capacity to export carbon through eCP is influenced by anthropogenic impact. The ROIs referring to these watercourses are identified in the geodatabase with the value of the attribute *FFR* equal to zero. Although the probabilistic river-driven forest loss model was developed to classify the level of impact of non-riverine activities in forest changes, the high level of anthropogenic alterations can make the result less reliable. For this reason, the analysis of these ROIs is intended only for general comparative purposes with respect to the behaviour of free-flowing rivers. We found that, on average, their eCE_*A*_ was 40% lower than free-flowing rivers.

## Technical Validation

To ensure the reliability of the developed database, we based the entire evaluation procedure exclusively on derived and validated satellite products (see section *Description of global datasets used in the study* and Table 1). A probabilistic approach was devised to evaluate the uncertainty surrounding the assessment of eCE. The aggregated assessment of continental eCE for the largest tropical rivers was derived by summing the computed values within each ROI across the continent. The uncertainty, denoted by the symbol *σ* and representing the standard deviation, can be calculated at the pixel level using principles from probability theory. The eCE consists of the product of two variables, both affected by errors: the river-driven forest loss area and biomass carbon density. Hence, these variables can be viewed as random processes. As outlined in the aforementioned filtering process, for each pixel, forest loss can be linked to a discrete random variable *χ*_*j, k*_ with two possible outcomes: 1 with a probability of *P*_*j, k*_ (RDFL) or 0 with a probability of 1-*P*_*j, k*_ (non-RDFL). This corresponds to a Bernoulli process^54^, akin to repeated coin flipping, where the mean is equal to *P*_*j, k*_ and the variance is equal to

12 $${\sigma }_{{\chi }_{j,k}}^{2}={P}_{j,k}\left(1-{P}_{j,k}\right).$$

The carbon density, on the other hand, is a continuous random variable with a mean of *ρ*_*j, k*_ (obtained from methods M1 to M4) and a standard deviation of ${\sigma }_{{\rho }_{j,k}}$. Employing Goodman’s expression^55^ for the variance of the product of two uncorrelated random variables, the error variance of the eCE for pixel (*j*, *k*) can be expressed as follows:

13 $${\sigma }_{{{\rm{eCE}}}_{j,k}}^{2}={A}_{j,k}^{2}{P}_{j,k}\left[{\sigma }_{{\rho }_{j,k}}^{2}+\left(1-{P}_{j,k}\right){\rho }_{j,k}^{2}\right],$$

where *A*_*j, k*_ is the pixel area. Per-pixel values for ${\sigma }_{{\rho }_{j,k}}$ are not explicitly provided in the raw datasets examined in this study. Therefore, we made use of various conservative assumptions. These assumptions are grounded in the observation that residuals are proportionate to the mean, as also suggested in ref. ^27^. Accordingly, in M1 and M2, we set

14 $${\sigma }_{{\rho }_{j,k}}={C}_{{\rm{v}}}\cdot {\rho }_{j,k},$$

with the coefficients of variation *C*_v_ ranging between 0.5 and 1.25. In M3 and M4, ${\sigma }_{{\rho }_{j,k}}$ was set to a constant value throughout the ROI, equal to the standard deviation of all the carbon densities measured inside the ROI, as reported in the *WHRC Carbon Stock* datasets^28^ for M3, and the dataset by ref. ^27^ for M4. As a further step, to advance in the analysis, the propagation of uncertainty from the pixel to the continental scale necessitates evaluating the spatial correlation of the errors. Neglecting this consideration may result in the cancellation of per-pixel errors and lead to a substantial underestimation of the overall uncertainty.

In this particular context, conventional utilization of spatial variograms (sense ref. ^27^), is impractical due to the spatial irregularity of Regions of Interest (ROIs), the heterogeneity of biomass caused by river dynamics, and the computational constraints, even when implemented in Google Earth Engine (GEE). Hence, following the approach outlined in^27^, we adopted two empirical autocorrelation length-scales (ALS), namely ALS_1_ set at 500 meters and ALS_2_ equivalent to the ROI area. It was conservatively assumed that pixels are perfectly correlated within a distance smaller than the ALS and uncorrelated beyond that distance. The dataset was partitioned into independent blocks using squares (for ALS_1_) or ROI polygons (for ALS_2_), and an upper conservative estimate of uncertainties was computed for each block by utilizing all the *σ*eCE*j, k* values obtained from Goodman’s formula within the block. For each method M1 – M4, the uncertainty in eCE at the continental scale, denoted as ${\sigma }_{cont,ALS}^{2}$, was calculated for both ALS values by summing the variance associated with each block within the continent: ${\sigma }_{cont,ALS}^{2}={\sum }_{i}^{N}{\sigma }_{i,ALS}^{2}$, where *N* is the number of blocks in a continent, and ${\sigma }_{i,ALS}^{2}$ is the variance error associated with each block. For methods M3 and M4, errors were assessed solely with ALS_2_, as in both scenarios, carbon density was derived from a spatial average at the ROI scale. Within each block, the variance was computed by determining its supremum over the block $(\mathop{sup}\limits_{(j,k)\in {\rm{block}}}\{{{\rm{eCE}}}_{j,k}\})$. By combining methods M1–M4 with the two autocorrelation length-scales and considering the four values of C_*v*_ for methods M1 and M2, a total of eighteen different configurations were examined for uncertainty assessment (refer to Table 5). The most conservative configuration (maximum uncertainty) yields a standard deviation (in TgC/yr) and percentage error of 0.84 (9.78%), 0.36 (16.53%), and 0.29 (24.57%) for South America, tropical Africa, and Asia/Oceania, respectively.

## Usage Notes

To allow an easy and visual representation of the eco-morphodynamic carbon processes throughout the tropical river network, the main results of the analysis are also visualized and disseminated through a WebGIS application available at the following link WebGIS.

From a technical point of view, the WebGIS application was created using the ESRI ArcGIS Instant Apps that enable the creation of web apps that make it easy to interact with maps and data, exploiting predefined templates to be customised to meet specific requirements. Firstly, both point and polygon feature classes were published on a server as web feature layers to be used in the WebGIS application. Secondly, starting from a basic template the WebGIS application has been customised to enhance map readability (in terms of symbology and geometries), easiness of use and relevance of displayed information.

**Fig. 9:**
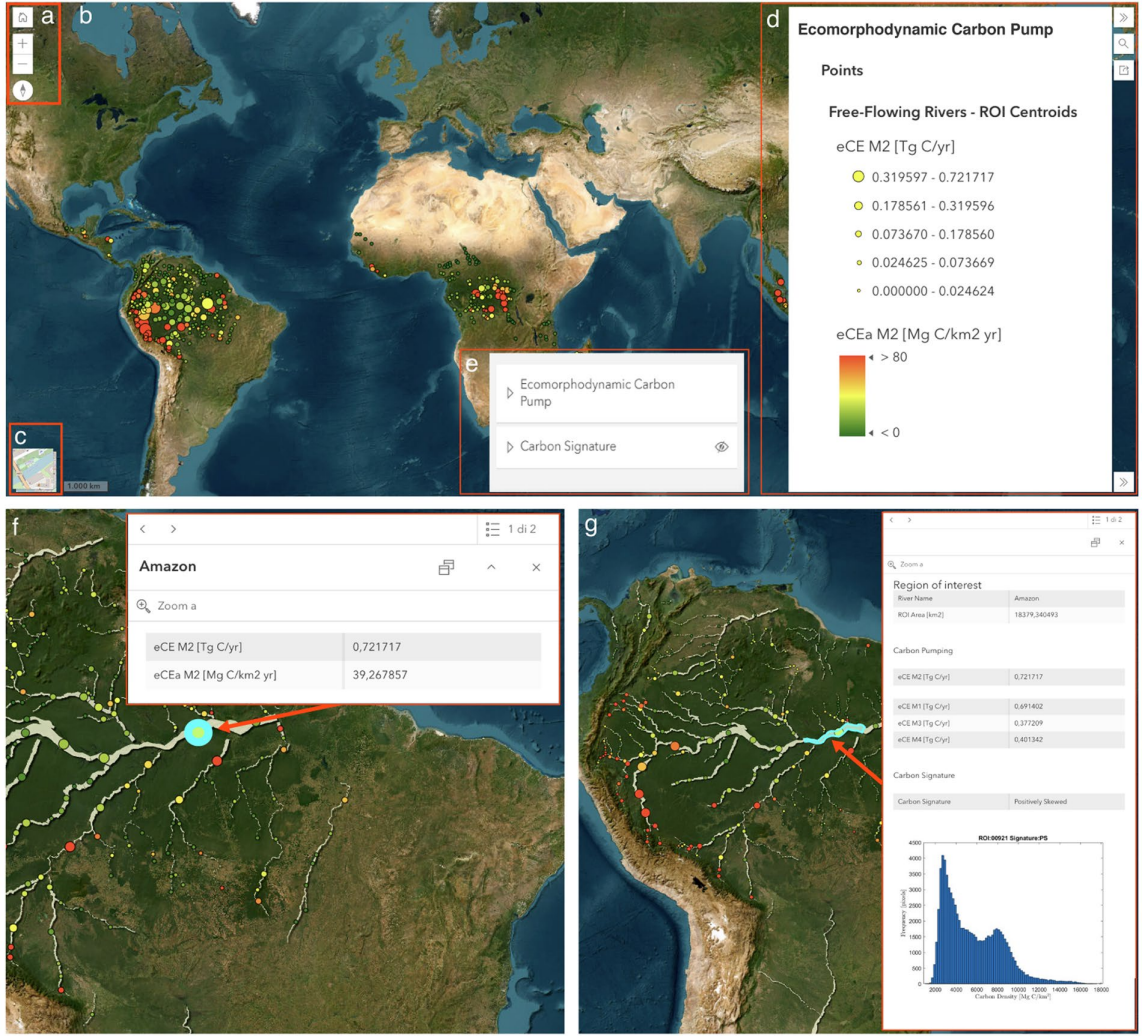
Graphical user interface of the WebGIS application illustrating the key findings of the study: (**a**) Navigation panel enabling users to set zoom levels and map orientation. (**b**) Data visualization: in the layer *Ecomorphodynamic Carbon Pumping* point sizes correspond to eCE, colours represent eCE_*a*_, and polygons denote regions of interest; in the layer *Carbon Signature* point sizes correspond to eCE, colours represent carbon signature classes. (**c**) Basemap with selectable options for open street map or satellite map views. (**d**) Legend explaining point size and colour codes for the point layers in the *Ecomorphodynamic Carbon Pumping* group. (**e**) Panel for selecting feature classes to display on the map, providing the option to show points representing centroids of regions of interest or polygons defining the ROIs. (**f**) Query results displaying information associated with centroids (eCE and eCEA). (**g**) Query results presenting information associated with polygons (ROI area, eCE M1-M4, carbon signature, and carbon biomass distribution within the ROI).

The WebGIS - mainly aimed at making both geographical and quantitative data easily accessible - is characterised by a main map where the point dataset is displayed with a bivariate symbology, i.e. in the layer *Ecomorphodynamic Carbon Pump* a colour palette related to eCE_*a*_ [Mg C/km2 yr], and the size proportional to eCE [Tg C/yr], in the layer *Carbon Signature* point sizes correspond to eCE [Tg C/yr], colours represent carbon signature classes. Selecting a point leads to a pop-up window displaying the parameters described before. Additionally, the polygon dataset is displayed at larger map scales (to avoid overcrowding the map) enabling the visualization of the ROIs and enriching the content of the related pop-up windows with additional parameters (a selection of the attributes described in Table 4) and the ROI’s actual carbon density distribution (as histogram bars). The user can easily display the layer legends, customize the layer visibility and choose between a satellite basemap or a cartographic basemap (based on OpenStreetMap data). The graphical user interface of the WebGIS application is shown in Fig. 9.

## Data Availability

The Java script for the GEE platform generating the row dataset, and the Matlab scripts generating the definitive data set are deposited in 10.6084/m9.figshare.24794295.v2, and are freely available.

## References

[1] Cole JJ (2007). Plumbing the global carbon cycle: Integrating inland waters into the terrestrial carbon budget. Ecosystems.

[2] Wohl E, Hall RO, Lininger KB, Sutfin NA, Walters DM (2017). Carbon dynamics of river corridors and the effects of human alterations. Ecol. Mono..

[3] Battin TJ (2008). Biophysical controls on organic carbon fluxes in fluvial networks. Nat. Geosci..

[4] Regnier P (2013). Anthropogenic perturbation of the carbon fluxes from land to ocean. Nat. Geosci..

[5] Evans, M. *Geomorphology and the carbon cycle* (Wiley, 2022).

[6] Drake TW, Raymond PA, Spencer RGM (2018). Terrestrial carbon inputs to inland waters: A current synthesis of estimates and uncertainty. Limnol. Oceanogr. Lett..

[7] Salerno L, Vezza P, Perona P, Camporeale C (2023). Eco-morphodynamic carbon pumping by the largest rivers in the neotropics. Sci. Rep..

[8] Vannote RL, Minshall GW, Cummins KW, Sedell JR, Cushing CE (1980). The River Continuum Concept. Canadian Journal of Fisheries and Aquatic Sciences.

[9] Ruiz-Villanueva, V., Piégay, H., Gurnell, A. A., Marston, R. A. & Stoffel, M. Recent advances quantifying the large wood dynamics in river basins: New methods and remaining challenges, 10.1002/2015RG000514 (2016).

[10] Guyette RP, Dey DC, Stambaugh MC (2008). The temporal distribution and carbon storage of large oak wood in streams and floodplain deposits. Ecosystems.

[11] Torres M (2017). Model predictions of long-lived storage of organic carbon in river deposits. Earth Surf. Dyn..

[12] Ghaffarian H (2023). Observer-bias and sampling uncertainties in riverine wood flux and volume estimation from video monitoring technique. Earth Surface Processes and Landforms.

[13] Ghaffarian H (2020). Video-monitoring of wood discharge: first inter-basin comparison and recommendations to install video cameras. Earth Surf. Process. Landf..

[14] Gurnell A, Piégay H, Swanson F, Gregory S (2002). Large wood and fluvial processes. Freshw. Biol..

[15] Holt DM, Jones EB (1983). Bacterial degradation of lignified wood cell walls in anaerobic aquatic habitats. Appl. Environ. Microbiol..

[16] Torres, J. A. Wood decomposition of Cyrilla racemiflora in a tropical montane forest. *Biotropica* 124–140 (1994).

[17] Junk, W. *The Central Amazon Floodplain* (Springer, 1997).

[18] Mackensen J, Bauhus J, Webber E (2003). Decomposition rates of coarse woody debris–a review with particular emphasis on Australian tree species. Aust. J. Bot..

[19] Archer, D. *The global carbon cycle* (Princeton University Press, 2010).

[20] Salerno L, Giulio Tonolo F, Camporeale C (2024). Figshare.

[21] Swanson ME (2011). The forgotten stage of forest succession: early-successional ecosystems on forest sites. Frontiers in Ecology and the Environment.

[22] Segatto PL, Battin TJ, Bertuzzo E (2023). A network-scale modeling framework for stream metabolism, ecosystem efficiency, and their response to climate change. Water Resour. Res..

[23] Grill G (2019). Mapping the world’s free-flowing rivers. Nature.

[24] Gorelick N (2017). Remote Sens. Environ..

[25] Allen GH, Pavelsky T (2018). Global extent of rivers and streams. Science.

[26] Hansen MC (2013). High-resolution global maps of 21st-century forest cover change. Science.

[27] Baccini A (2012). Estimated carbon dioxide emissions from tropical deforestation improved by carbon-density maps. Nat. Clim. Change.

[28] Zarin DJ (2016). Can carbon emissions from tropical deforestation drop by 50% in 5 years?. Glob. Change Biol..

[29] Pekel J-F, Cottam A, Gorelick N, Belward AS (2016). High-resolution mapping of global surface water and its long-term changes. Nature.

[30] Giglio L, Justice C, Boschetti L, Roy D (2015). NASA EOSDIS Land Processes DAAC.

[31] Linard, C., Gilbert, M., Snow, R. W., Noor, A. M. & Tatem, A. J. Population distribution, settlement patterns and accessibility across Africa in 2010. *PLoS ONE* **7**, 10.1371/journal.pone.0031743 (2012). 10.1371/journal.pone.0031743 PMC3283664 22363717

[32] Gaughan, A. E., Stevens, F. R., Linard, C., Jia, P. & Tatem, A. J. High resolution population distribution maps for Southeast Asia in 2010 and 2015. *PloS one* **8**, 10.1371/journal.pone.0055882 (2013). 10.1371/journal.pone.0055882 PMC3572178 23418469

[33] Sorichetta A (2015). High-resolution gridded population datasets for Latin America and the Caribbean in 2010, 2015, and 2020. Sci. data.

[34] Friedl, M. & Sulla-Menashe, D. MCD12Q1 MODIS/Terra + aqua land cover type yearly L3 global 500 m SIN grid V006 [data set]. *NASA EOSDIS Land Processes DAAC* **10**, 10.5067/MODIS/MCD12Q1.061 (2015).

[35] Linke S (2019). Global hydro-environmental sub-basin and river reach characteristics at high spatial resolution. Sci. Data.

[36] Gurnell A (2016). A multi-Scale Hierarchical Framework for Developing Understanding of River Behaviour to Support River management. Aquat. Sci..

[37] Rodriguez-Iturbe, I. & Rinaldo, A. *Fractal river basins: chance and self-organization* (Cambridge University Press, 2001).

[38] Schöngart, J. & Wittmann, F. Biomass and net primary production of central Amazonian floodplain forests. In *Amazonian Floodplain Forests*, 347–388 (Springer, 2010).

[39] Venter O (2016). Sixteen years of change in the global terrestrial human footprint and implications for biodiversity conservation. Nat. Commun..

[40] Lloyd CT (2019). Global spatio-temporally harmonised datasets for producing high-resolution gridded population distribution datasets. Big earth data.

[41] Jones MW (2020). Fires prime terrestrial organic carbon for riverine export to the global oceans. Nat. Commun..

[42] Paine ADM (1985). Ergodic reasoning in geomorphology: time for a review of the term?. Prog. Phys. Geogr..

[43] Saatchi SS (2011). Benchmark map of forest carbon stocks in tropical regions across three continents. Proc. Nat. Acad. Sci..

[44] Salerno, L. *et al*. Satellite analyses unravel the multi-decadal impact of dam management on tropical floodplain vegetation. *Front. Environ. Sci*. **357** (2022).

[45] Camporeale C, Perucca E, Ridolfi L, Gurnell A (2013). Modeling the interactions between river morphodynamics and riparian vegetation. Rev. Geophys..

[46] Muneepeerakul, R., Rinaldo, A. & Rodriguez-Iturbe, I. Effects of river flow scaling properties on riparian width and vegetation biomass. *Water Resour. Res*. **43**, 10.1029/2007WR006100 (2007).

[47] Salo J (1986). River dynamics and the diversity of Amazon lowland forest. Nature.

[48] Constantine JA, Dunne T, Ahmed J, Legleiter C, Lazarus ED (2014). Sediment supply as a driver of river meandering and floodplain evolution in the Amazon Basin. Nat. Geosci..

[49] Hess LL, Melack JM, Novo EMLM, Barbosa CCF, Gastil M (2003). Dual-season mapping of wetland inundation and vegetation for the central Amazon basin. Remote Sens. Environ..

[50] Lima AJN (2012). Allometric models for estimating above-and below-ground biomass in Amazonian forests at São Gabriel da Cachoeira in the upper Rio Negro, Brazil. For. Ecol. Manag..

[51] Dargie GC (2017). Age, extent and carbon storage of the central Congo Basin peatland complex. Nature.

[52] Morel AC (2011). Estimating aboveground biomass in forest and oil palm plantation in Sabah, Malaysian Borneo using ALOS PALSAR data. Forest Ecol. Manag..

[53] Margono BA, Potapov PV, Turubanova S, Stolle F, Tang MC (2014). Primary forest cover loss in Indonesia over 2000–2012. Nat. Clim. Change..

[54] Ross, S. *Probability and statistics for engineers and scientists* (Elsevier, New Delhi, 2009).

[55] Goodman LA (1960). On the Exact Variance of Products. J. Am. Stat. Assoc..

[56] Chambers JQ, dos Santos J, Ribeiro RJ, Higuchi N (2001). Tree damage, allometric relationships, and above-ground net primary production in central Amazon forest. Forest Ecol. Manag..

