# Author Correction: A global dataset of carbon pumping by the world’s largest tropical rivers

**DOI:** 10.1038/s41597-024-03516-5

**Published:** 2024-06-19

**Authors:** Luca Salerno, Fabio Giulio Tonolo, Carlo Camporeale

**Affiliations:** 1 DIATI Department of Environment, Land and Infrastructure Engineering, Politecnico di Torino, Turin, 10129 Italy; 2 DAD Department of Architecture and Design, Politecnico di Torino, Turin, 10129 Italy

**Keywords:** Environmental impact, Hydrology, Environmental impact, Hydrology

Correction to: *Scientific Data* 10.1038/s41597-024-03201-7, published online 13 April 2024

In this article the funding from ‘the e-Capture project – funded by the European Union – Next Generation EU within the PRIN 2022 program (D.D. 104 - 02/02/2022 Ministero dell’Università e della Ricerca)’ was omitted. The original article has been corrected.

